# Toward a knowledge-synthesis heuristic for sport leaders: the strategic leader synthesis model

**DOI:** 10.3389/fspor.2024.1408887

**Published:** 2024-06-27

**Authors:** John Cairney, Veronique Richard, David Legg

**Affiliations:** ^1^ Queensland Centre for Olympic and Paralympic Studies, The School of Human Movement and Nutrition Sciences, The University of Queensland, St Lucia, QL, Australia; ^2^ Faculty of Health and Physical Education, Mount Royal University, Calgary, AB, Canada

**Keywords:** leadership, sport management, roles, capabilities, mindset, strategic leader

## Abstract

Sport management leadership research has predominantly focused on leadership behaviours, particularly transformative leadership, without fully acknowledging the complex, multifaceted nature of leadership within the sports context. This perspective overlooks the reality that sports leaders operate within complex organizations and varied contexts that significantly influence their behaviours. Leadership in sports demands core capabilities in decision-making, communication, and strategic thinking, and a mindset that influences perception, decision-making, and behaviour. Consequently, a singular focus on transformative leadership may undervalue the importance of other attributes. This paper thus argues for a comprehensive leadership framework that integrates behaviours, roles, capabilities, and mindset, and draws insights from business management. By proposing this framework organized across four domains—Context, Roles, Capabilities, and Mindset—this paper aims to foster a deeper understanding of sports leadership dynamics, highlighting the necessity of a holistic approach that considers the interconnectedness of these elements.

## Introduction

Leadership research in sport management has tended to focus on leadership behaviours, the most common being transformative (1). While this approach has merits, it fails to consider the multifaceted nature of leadership in sport. Leaders, be they coaches, players or administrators, often work inside complex organisations, and in varied contexts, which directly influences the required leadership behaviour (2). Leaders hold formal and informal roles and are often required to shift those depending on context and specific circumstances (3). Sport leadership also requires core capabilities, in decision-making, communication and strategic thinking. Finally, mindset, or ways of thinking, influence how leaders perceive the context in which they work, the decisions they make, and the behaviours they enact (4). The emphasis on a single leadership style, such as that found in transformative leadership, may overshadow the importance of these varied and foundational skills and the role they play in effective leadership. Consequently, a more comprehensive leadership framework is proposed which considers behaviours, roles, capabilities, and mindset.

In their comprehensive review of leadership scholarship within sport management, Welty Peachey et al. (1) also highlighted the narrow focus of leadership studies and they too proposed a conceptual framework that linked individual antecedents (e.g., “darker” traits) to leadership outcomes. Their framework is situated within a multi-level model that builds upon the work of Yammarino (5) and while this model represented an advancement in leadership theory by moving beyond single-level approaches, in our opinion it focused mostly on context and overlooks the significant interplay between roles, capabilities, and mindset. Although these latter factors arguably still operate at the behavioural level, they offer a nuanced understanding of leadership dynamics, suggesting a shift away from focusing solely on discrete leadership behaviours.

In this paper, we hope to further the understanding of leadership in sport by introducing a preliminary framework derived from the mainstream literature on leadership in business management. As Welty Peachey et al. (1) note, much of the leadership research in sport management incorporates models and concepts from the fields of business and psychology. While this observation is accurate, the relatively limited translation of leadership knowledge within the sport context suggests that sport management has not fully capitalized on the wealth of contemporary leadership theories available (6–8). Our approach, therefore, aims to extract insights from business management and psychology to establish a framework that facilitates greater understanding of the complexities and multi-faceted nature of leadership. This way, insights can be used to further establish sports as a unique context for examining these theories (1). For example, athletes and coaches will have a particular influence on leadership on the management and business side of professional teams, creating power dynamics that are specific to the sporting context (1). Importantly, this framework aims at translating business leadership practices to facilitate the leadership endeavours of professional, amateur high-performance, and sport industry contexts (e.g., marketing, communications, sport tech) with the caveat that it may not be contextually suited to on-field sport leadership. Indeed, the models we draw from (9–11) are not specific to an organizational level, industry or particular context. This certainly does not imply that all aspects of the framework apply equally to all contexts. The leaders' key tasks (e.g., administrative vs. technical), the context they work in (professional vs. amateur sport) including the size and complexity of the organisation, will all influence which aspects of the framework are most salient. Nonetheless, many of the concepts discussed may still find relevance with leaders across various types of sport organizations, as well as with coaches. Wherever a leader is responsible for others (e.g., teams, be they workers or athletes), the application of the framework, in whole or part, may be useful.

**Figure 1:**
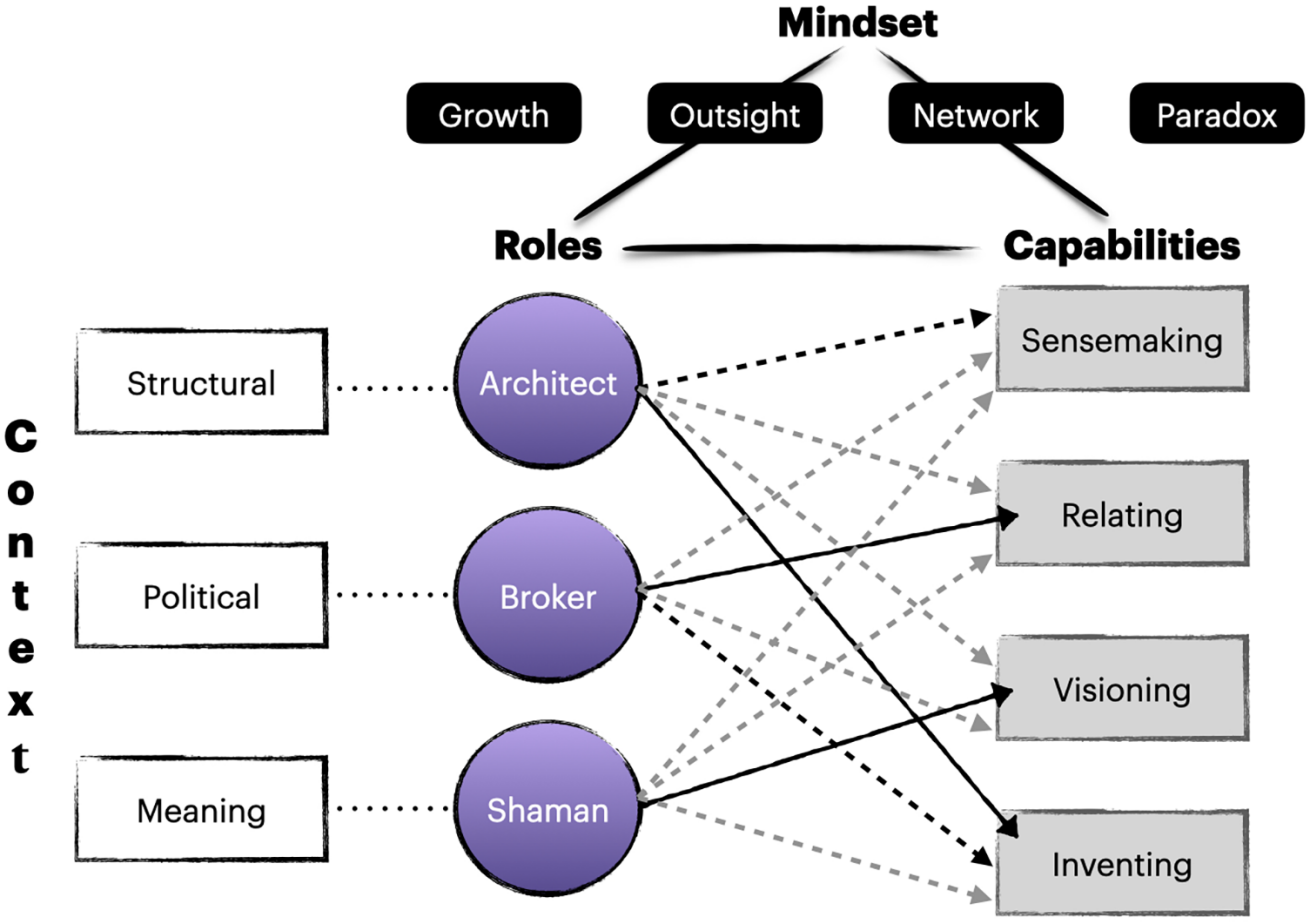
The strategic leader synthesis model.

The framework (see Figure 1) we propose is organized across four domains: Context, Roles, Capabilities, and Mindset. The three (originally four) frames model (11) is used to analyse organisational contexts and the actions of leaders within them by considering the interplay among structure (e.g., policies, formal processes, roles), politics (organisations as political arenas), and meaning—how meaning is constructed and reconstructed inside organisations and the role of leaders in shaping organisational narratives.

The leadership styles used in this approach include *architect*, *broker,* and *shaman* and these are mapped onto structure, politics and meaning respectively, and come directly from Bolman and Deal (11). Acting as a broker, for example, highlights a political role of the leader and the necessity at times, to build coalitions and/or negotiate across diverse stakeholders. The architect role is about building structures and processes to facilitate action. It is structural in the sense that it is about creating conditions for success in an organisation. Shaman, meanwhile, emphasizes the importance of a leader being seen to be driven by purpose based on a vision for a future success. In this way, a leader provides meaning and purpose to an organisation's existence.

The novel aspect of our strategic leader framework lies in its attempt to integrate insights from other perspectives into the modified three frames model, thus forming a synthesis of concepts and insights. For example, leadership capabilities are derived from the 4-CAP model (9, 10), which emphasizes the essential capabilities for effective leadership in complex environments. These capabilities include sensemaking, relating, visioning, and inventing.

Sensemaking is about understanding the context, internally and externally, and using that information to formulate planning. It is a continual process, where a leader is constantly asking, how will the changes in our environment influence us now and into the future? Relating is about building trust. It involves a balance of inquiry with advocacy (10), which requires both effective listening skills and the ability to constructively communicate ideas to followers. Visioning is the ability to communicate a future state that brings others along while inventing is the creation of new ways of doing, of experimenting and learning from mistakes. It is fundamentally about creating a learning culture—a place where experimentation and risk are encouraged, failure is viewed as a learning opportunity, and professional growth and development is encouraged and supported. Together, Ancona and colleagues (10) argue that this is how leaders build credibility.

The arrows in Figure 1 illustrate the connections between the three roles (architect, broker and shaman) and the four capabilities identified in the 4-CAPS+ model (9, 10). The dark arrows emphasize the strongest and most apparent links (e.g., shaman role is strongly connected to vision capability), the black dotted arrows emphasise a moderately strong connection with a second capability, while the grey, dotted arrows indicate that each role draws at least partially (or weakly) on each capability. For instance, for a shaman to be successful at visioning, they must also be capable relators and sense makers.

Looking at the multiple contexts of a National Sport Organisation (NSO), the Executive General Manager (EGM) a few months prior to the 2024 Paris Olympic and Paralympic Games provides a relevant example of the interactions between these roles and capabilities. First, months before the Games, the EGM might have to build coalitions with other countries (i.e., broker role) by establishing trust with the main stakeholders (i.e., relating) to organise a common staging camp aiming at finalising the preparation of athletes (e.g., team sports). In parallel, the EGM might also be designing the strategic plan for the next quadrennial (i.e., inventing and sensemaking) and presenting it to investing partners to secure future funding (i.e., architect role). All this is being done while promoting a vision to inspire the current Olympic and Paralympic teams (i.e., visioning) to perform at their best (i.e., shaman role).

Overarching these contextual roles and capabilities, the final domain is *mindset*, which aims to capture broad meta-orientations, required to lead in complex environments. It draws from numerous sources. Within the domain of mindset is a focus on growth, which is the belief that intelligence and abilities can be developed through dedication, effort, and resilience. Individuals with a growth mindset perceive challenges as opportunities for growth, view effort as a path to mastery, and embrace setbacks as learning experiences (12). By promoting a growth mindset culture within organizations, leaders can encourage innovation, continuous learning, and a supportive environment that values effort and improvement (13). In other words, a growth mindset is useful for resilience at the leader level, but also for creating optimal environments for performance, creativity, and innovation.

Ibarra's (14) work on the importance of outsight is also an inspiration for this domain. Outsight emphasizes action as essential to becoming (i.e., to become a shaman, one must first act the part). Ibarra suggests that to develop as leaders, individuals must step outside their comfort zones, engage with a broad range of people and experiences, and experiment with new roles and activities. The exposure to diverse perspectives and contexts then enables leaders to expand their understanding, rethink assumptions, and approach problems and opportunities in novel ways. Outsight is particularly crucial in fast-paced, ever-changing business environments, where adaptability and creativity are key to success. Through outsight, leaders can better navigate complexity, drive change, and inspire others, leveraging external insights to enhance strategic thinking and decision-making.

The third critical component of mindset emphasizes the significance of networks, both as a tool for mapping connectivity through social network analysis among individuals within organizations and with external entities (15, 16). Networks are crucial for the strategic development of leadership capabilities, enabling leaders to expand or challenge perspectives, gain new insights or crucial information, forge alliances (17), and bridge gaps between groups (14) and stakeholders (16). Moreover, networks offer opportunities for mentorship and role modelling, which are vital for the transformation of professional identity (14). For example, a football coach could benefit from the insights of a basketball coach on managing star athletes, or a swimming coach's strategies for individual performance optimization. Additionally, by bridging networks between sports medicine professionals and sports psychologists, a coach can create a holistic support system for their athletes, enhancing both physical and mental resilience. Such interconnectivity not only enriches the coach's leadership capabilities but also fosters a culture of innovation and continuous.

Lastly, the concept of paradox is rooted in the understanding that organizations often face trade-offs between competing or conflicting priorities, referred to as “strategic paradoxes” (18). To navigate these effectively, organizations must establish guardrails to prevent power imbalances among competing groups. This framework does that by ensuring that leaders engage in dynamic decision-making, a critical factor for achieving equilibrium within the organization (18). Furthermore, leaders are encouraged to adopt a “both/and” mindset (19), which allows them to manage tensions arising from paradoxes sufficiently long enough to enable thorough decision-making, thereby creating a conducive environment for balancing exploitation (optimizing current resources) and exploration (innovating for the future). General managers for example often face a critical decision at the trade deadline: whether to trade future draft picks and young prospects for a star player who could immediately improve the team's performance and increase its chances of winning a championship in the short term. This decision embodies the paradox between the immediate need to exploit current opportunities for success and the long-term strategy of exploring and developing young talent that could secure the team's future competitiveness. Crucially, for a leader to thrive, they must welcome paradox, be invigorated by it, rather than overwhelmed (20).

The strategic leader framework we propose thus progresses from context and roles, to the specific capabilities required of those roles, and to over-arching mindsets (attitudes and beliefs that shape perception and provide an interpretative lens for understanding the world around us), which influence the practice of those capabilities and therefore, behaviour inside roles. The triangle at the top of Figure 1 represents the intricate connections between these domains. Pragmatically, this gives the leader a framework from which to reflect on their own practice and the practices of others. What role I am playing? What role is needed? What capabilities should I be engaging in and how can I use outsight, networking, and paradoxical thinking to achieve goals? The literature from which this heuristic is derived can then also serve as fodder for deeper dives into each of the domains, encouraging a journey of learning and discovery. However, it is critically important to emphasize that no leader will be able to enact all of these elements or have all the capabilities required across all domains. It is the ideal, something to strive toward. It also can orient leaders to what they need to draw from others.

### An application of the strategic leader framework: Sir Alex Ferguson

To illustrate the utility of the framework, we have applied our model to a well-known figure in sport, Sir Alex Ferguson, former manager of the Manchester United Football Club. There is strong evidence of Ferguson's acumen as a shaman. From very early in his tenure, he had a vision for sustained success through modernisation of the club's youth development system and an unconventional belief that it was possible to win with young players (21). Ferguson also showed great adeptness in his role as an architect, not only through the creation of a system that developed Ryan Giggs, Paul Scholes and David Beckham, but through his reputation as a tactician, demonstrating flexibility and innovation to game plans. Achieving by-in to his systems then required skills as broker, working with coaches, players and ownership to successfully implement his vision.

Through these roles, we recognize the implementation of those core leadership capabilities needed for success. Ferguson was described as a “portfolio manager” (21) of talent, which means he was highly effective at *sensemaking*—understanding data and information to make decisions. Ferguson was also highly respected by players, coaches, and other members of the club. Even though he set very high standards and often found himself in conflict, he earned respect as a highly effective and trustworthy leader. In other words, through his unique style, he was able to *relate* to others and this helped forge the path needed to realize his vision. As noted already, Ferguson was also proficient at *visioning* and *inventing* as demonstrated by his unique strategies and development systems.

In terms of mindset, we also see evidence consistent with our framework in Ferguson's leadership style. Over his career, he assembled five unique championship teams, in part, because he understood the importance of cycles, in players career's and at an organisational level. To be successful, he needed to build a team that could win today and also in the future. Short-term and long-term objectives were not viewed as competing (either/or) but complementary (both/and) reflecting his *paradoxical mindset* (22). It is also evident that Ferguson had a system thinking (i.e., *network mindset*) approach. His investment in player development, team dynamics, and tactical strategies, gave him a holistic perspective that allowed him to make decisions that considered the broader impact of overall performance to the success of the club. Finally, we see Ferguson use *outsight* to gain new perspectives that influenced his leadership. In 2002, he retired for a brief period, and this allowed him to re-assess his role, learn from the experience of stepping out, and return with renewed insights. In taking this decisive action, Ferguson clearly saw the valuing of acting, not just reflecting or thinking. He needed to take a bold step and transform his role, to acquire new perspective (23).

Of course, Ferguson is a truly exceptional example of leader, one who was adept at many of the elements found in our framework. But he is not representative of most leaders, either in sport or in other contexts. Indeed, Ferguson's own reflections on his leadership suggest he did not come ready made with these abilities. He learnt, adapted, and grew in his role over time, which is very much consistent with the importance of creating learning cultures within organisations (10). The space to evolve as a leader is as important as providing growth opportunities for followers. We chose Ferguson to illustrative the inter-connectivity and relatedness of the core domains of the framework, but the intent of the framework is certainly not to suggest that to be a successful leader, one must be adept at ALL the capabilities and attributes each contains. As Ancona and her colleagues argue (10), most leaders are incomplete, and their strengths defined by competences in one or two of those core capabilities. Perfection is not the ideal, however, successful leaders recognise both their strengths and limitations. They surround themselves with others who have the capabilities they lack and, they know when to step back and let others lead. Despite his broad talents and capabilities, Ferguson practiced letting go of control when he needed it the most:

“One afternoon at Aberdeen I had a conversation with my assistant manager … He said, “I don’t know why you brought me here.” … “I don’t *do* anything. I work with the youth team, but I’m here to assist you with the training and with picking the team. That's the assistant manager's job.” … At first, I said, “No, no, no,” but I thought it over for a few days and then said, “I’ll give it a try. No promises.” Deep down I knew he was right. So, I delegated the training to him, and it was the best thing I ever did.” (21)

The point of the framework is thus not only to identify the contextual roles, capabilities, and mindsets of effective leaders so individuals can develop those elements, but it is also to identify where there may be gaps. Our suggestion is then to use the framework to think broadly about leadership and strategy, identify one's own strengths, but also what might be needed and achieved through building effective high performing teams, and empowering leadership in others.

### Future work

While most of the research in sport leadership has concentrated on transformative leadership (1), other areas have also been explored, such as gender differences (24) and leader-member exchange (25). In terms of leadership styles, ethical (26), servant (27), and authentic (28) approaches have also been considered within the sports context. Further research could connect these explorations by examining for instance how the core domains of our framework might be influenced by gender. Because our framework includes a paradoxical mindset, future research could also focus on embracing the tension between conflicting demands and adopting a “both/and” rather than an “either/or” approach to leadership (19), laying the groundwork for paradoxical leadership behaviours. Paradoxical leaders, drawing on eastern philosophy, navigate contradictions such as balancing closeness with followers while maintaining distance, combining control with autonomy, and promoting uniformity while valuing individualism (29). In our framework, it is this paradoxical mindset that underpins specific behaviours, which can manifest through various roles and across different capabilities within organizations. Future studies could thus explore other mindsets linked to leadership styles, such as transformative and/or servant-based approaches.

We deliberately focused on insights derived from mainstream business management and leadership literature, despite observations that the transfer of organizational behaviour theories to sport management is often limited (6–8). Chalip (30) has criticized this derivative approach, advocating instead for theories rooted specifically in the context of sport. Future research should thus delve into how contexts, roles, capabilities, and mindsets are shaped by the unique aspects of sport, including the influence of fans, alumni, coaches, and celebrity athletes (1), further refining the domains outlined. Developing these sport-specific factors in conjunction with our synthesis framework is crucial.

## Conclusion

Our paper pursued two principal objectives. First, in line with previous scholars (1, 31), our goal was to transcend examining single leadership behaviours/styles, focusing instead on the dynamic and highly contextualized process. This included exploring the interconnections among contexts, roles, capabilities, and mindsets within complex organizations. Secondly, we sought to integrate concepts and insights from mainstream literature on leadership and business management, applying these to leadership in sport. The resultant framework is heuristic, effectively weaving these diverse ideas into a cohesive visual that highlights the interconnectedness of the concepts. Ultimately, the model proposes that effective leadership necessitates leveraging all these elements, with each situation or challenge demanding a unique blend of roles, capabilities, and mindsets. Echoing the sentiments of other scholars, we embrace the concept of the “incomplete leader,” acknowledging that few leaders excel in all areas equally (10). A key aspect of effective leadership involves knowing when to involve others who possess the skills and abilities the leader lacks. Thus, the framework should be seen not as a tool for evaluating leadership efficacy but as a guide to help researchers and practitioners appreciate the wide array of domains that constitute effective leadership. Is the framework complete? Just as we embrace the notion of “incomplete leader,” we acknowledge that heuristic frameworks are “works in progress.” It is precisely these limitations that underscore the need for further theorizing and conceptual development. As we saw in the case of Ferguson, although he was exceptional in embodying many elements of the framework, he did not come to Manchester “ready-made.” He had the opportunity to learn and develop as a leader. We discuss this in relation to creating learning cultures within organizations (10). Organizations that embrace experimentation and accept failure as an opportunity for growth create spaces for leaders and followers to develop. We are, of course, not ignoring the high-pressure stakes of competitive sports. Failure in business and failure in sports can have quite different consequences. For example, managers in professional clubs are often dismissed for “failing” to deliver “wins,” even if they are part of a complex system and thus cannot be held solely accountable.

This framework is introduced to foster broader awareness and dialogue regarding the complex interplay of contexts, roles, capabilities, and mindsets in sport leadership. Although it is not a finalized model and much synthesis remains, initiating discussions and validations of the concepts and insights for this framework with leaders in the sport industry is vital. For instance, focus groups with leaders from various sectors could critically assess these concepts and determine their resonance with sports leaders. This could also facilitate further exploration of how sport-specific contextual factors influence both the framework and leadership more broadly, guiding ongoing development and refinement. Specific elements within the framework would also be the focus of future research. For example, the conditions under which a “both/and mindset” might be most effectively used in the context of sport would be a novel area of inquiry in the sport management literature. This could be approached qualitatively (e.g., an ethnographic study of a “both/and mindset” in everyday interactions between managers, coaches, and players) or quantitatively [e.g., experiments involving scenarios (vignettes) to identify the conditions under which such a mindset might influence decision-making for sport leaders]. Ultimately, we hope that our endeavour lays the groundwork for a more nuanced understanding of effective leadership within the dynamic and multifaceted context of sport.
